# A context-based model of collaborative inhibition during memory search

**DOI:** 10.1038/s41598-024-78517-w

**Published:** 2024-11-12

**Authors:** Hemali Angne, Charlotte A. Cornell, Qiong Zhang

**Affiliations:** 1 Department of Computer Science, Rutgers University-New Brunswick, Piscataway, 08854 USA; 2 Department of Psychology, Rutgers University-New Brunswick, Piscataway, 08854 USA; 3 Rutgers Center for Cognitive Science, Piscataway, 08854 USA

**Keywords:** Collaborative inhibition, Group recall, Memory search, Computational modeling, Long-term memory, Human behaviour

## Abstract

Contrary to common intuition, a group of people recalling information together remembers less than the same number of individuals recalling alone (i.e., the collaborative inhibition effect). To understand this effect in a free recall task, we build a computational model of collaborative recall in groups, extended from the Context Maintenance and Retrieval (CMR) model, which captures how individuals recall information alone. We propose that in collaborative recall, one not only uses their previous recall as an internal retrieval cue, but one also listens to someone else’s recall and uses it as an external retrieval cue. Attending to this cue updates the listener’s context to be more similar to the context of someone else’s recall. Over an existing dataset of individual and collaborative recall in small and large groups, we show that our model successfully captures the difference in memory performance between individual recall and collaborative recall across different group sizes from 2 to 16, as well as additional recall patterns such as recency effects and semantic clustering effects. Our model further shows that collaborating individuals reach similar areas in the context space, whereby their contexts converge more than the contexts of individuals recalling alone. This convergence constrains their ability to search memories effectively and is negatively associated with recall performance. We discuss the contributions of our modeling results in relation to previous accounts of the collaborative inhibition effect.

## Introduction

Past work has long recognized the important role of context in the encoding and retrieval of information. Among the vast number of experiences that we can call to mind, it is commonly believed that a task-relevant context is used to guide recall, highlighting a subset of possible memories^1–5^. People remember more information when tested in their original environmental context than in a new one^6,7^. Context can also be endogenous, slowly changing over time and binding with encountered experiences. As the context similarity between study and test decreases, forgetting increases. This notion of context has been able to explain a wide range of memory phenomena in tasks of recognition^5,8^, cued recall^9^, free recall^4,10,11^, as well as reconciled alternative theories in serial recall^12^. Despite the influence of the context-based account in studying the memory of individuals, we do not yet know if it also accounts for recall behavior when a group of individuals collaborate together. The goal of the current work is to propose context as a unifying theory across individuals and groups.

**Figure 1:**
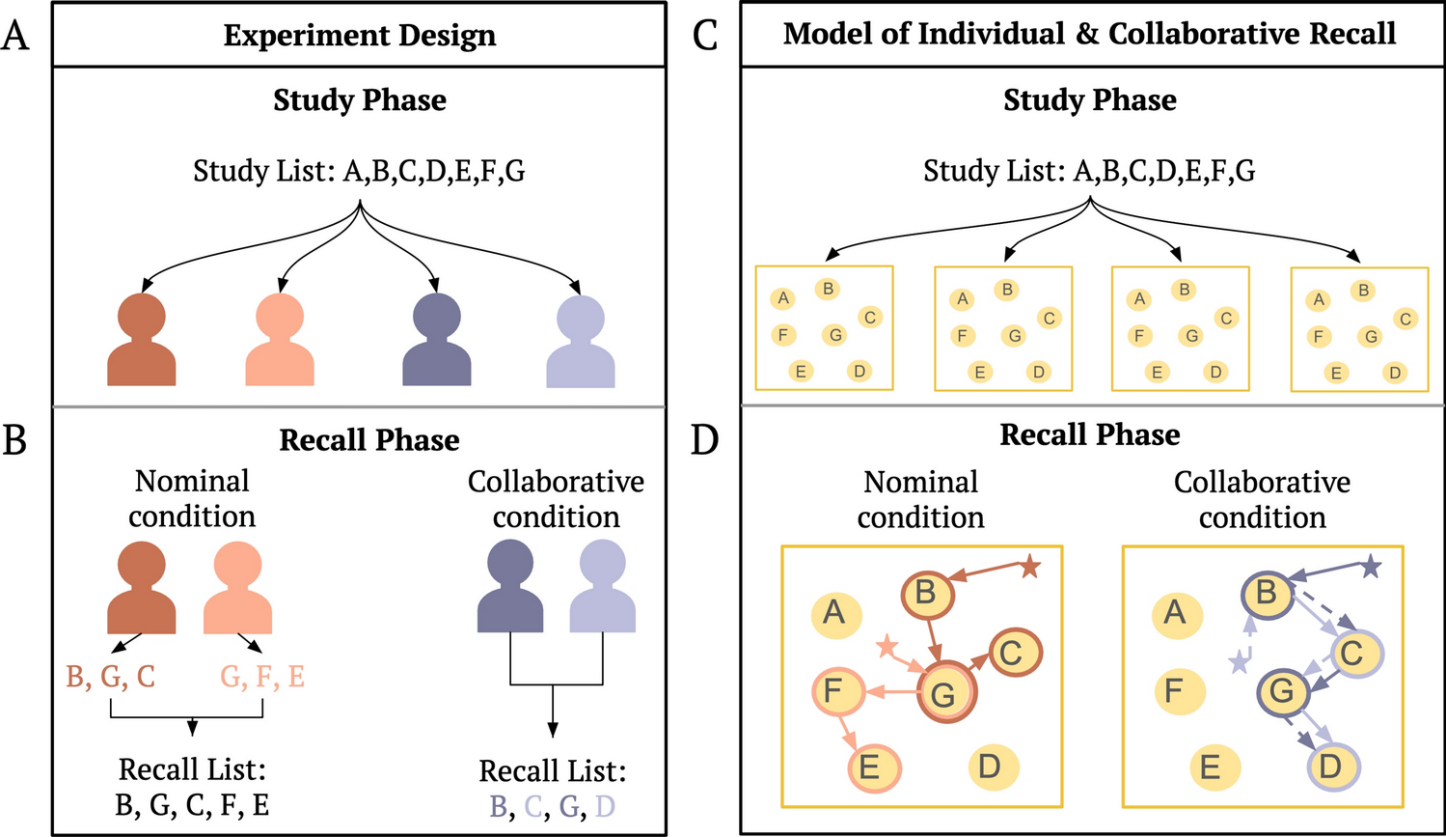
Experimental design of free recall in groups and our model of individual and collaborative memory search. (**A**) In the experiment, participants within a group learned the same list of items, and the presentation order was randomized for each individual (with individuals in the nominal and collaborative conditions paired). (**B**) Participants then recalled the list either alone (nominal condition members) or together (collaborative condition members). (**C**) In our model, participants encoded the list of items in a memory space to simulate the study phase. (**D**) To simulate the recall phase, nominal condition members searched this space independently using only their own recalls as internal retrieval cues (indicated by solid arrows). In the collaborative condition, members took turns recalling, using their own recalls as internal retrieval cues (indicated by solid arrows) and additionally having the chance to attend to others’ recalls as external retrieval cues (indicated by dashed arrows).

In our daily lives, where we spend the majority of our time in social contexts (e.g., classroom, family dinners, work meetings), we often recall information in a group instead of in isolation. While collaborative memory has largely been studied in the domains of sociology and social psychology^13–15^, more recently, cognitive research has begun to identify phenomena as well as mechanisms of how the memory of an individual changes when interacting with others in a group setting^16,17^. A counter-intuitive finding in this literature is the collaborative inhibition effect, where one’s memory becomes worse instead of better when remembering with others. In these studies (Fig. 1A, B), individuals perform a free recall task^18–20^ in which they each study a list of items. Then, they are asked to recall them in any order, either alone (nominal condition) or in a group (collaborative condition); surprisingly, the collaborative groups recall fewer items than individuals in the nominal condition (the summed recalls by the same number of individuals after excluding duplicates)^21–25^. The collaborative inhibition effect has been robustly observed across a range of empirical situations (for reviews, see Marion et al.^26^ and Rajaram et al.^16^).

Why does a collaborative group recall less information than the same number of individuals recalling individually? We propose that the collaborative inhibition effect is an emergent property of a context-based account. While the context of an individual in the nominal condition evolves on its own during recall, we hypothesize that the context of an individual in the collaborative condition is additionally influenced by the context of other individuals. Without introducing additional mechanisms, we will show that the collaborative inhibition effect naturally emerges from the interaction of individuals’ mental contexts as they recall information together. This provides a more parsimonious explanation of the phenomenon compared with several other accounts proposed in the past^24,27–29^.

To test that a context-based account can explain the collaborative inhibition effect, we build a computational model of individual recall and collaborative recall and compare our model’s behavior to an existing dataset of nominal and collaborative recall^22^. Figure 1 illustrates how we simulate our proposed model corresponding to their experimental paradigm. We achieve this by extending the Context Maintenance and Retrieval (CMR) Model, previously developed to capture an individual’s behavior in a free recall task^4,30–32^. According to the CMR model, experiences (items A-G) are organized into a latent context space based on their study order and semantic meanings (Fig. 1C). This process is the same across individuals in the nominal condition and the collaborative condition, as they completed the same procedure during study. Later at recall, how well these experiences can be retrieved depends on how close one’s current context location (indicated by a star in Fig. 1D) is to their study contexts. As the memory search process evolves, each new recall is driven by the context induced by the last recall. For example, in Fig. 1D, the dark orange individual recalling item B brings their current context closer to B in the context space, so the next recall will likely be an item close to B (such as G). Individuals in the nominal condition search this context space independently, which can be modeled by simulating multiple CMR models separately. To model behavior in the collaborative condition, we similarly assume that collaborating individuals always update their context following their own recalls. Additionally, we incorporate a new process to the standard CMR model to describe how listening to others’ recalls affects one’s own recall: whenever a person recalls an item, all other individuals in the collaborative condition update their context to this item with a probability of $p_{cue}$. For example, in Fig. 1D, after the purple individual recalled item B, the lilac individual listened to it and updated their context towards item B, leading the next recall to likely be close to item B (such as C). Consequently, in the collaborative condition, an individual’s recalls are driven both by the context of their own recalls and the context of others’ recalls.

Formulated this way, as recall unfolds, each collaborative member’s recall starts to converge to the contexts of others’ recall compared with that in the nominal condition. We hypothesize that this could be the key mechanism that contributes to the collaborative inhibition effect. Consider memory search in a context space analogous to foraging mushrooms in a forest. When individuals explore their memory (or a forest) independently, they can explore unique areas as only their own retrieval of memories (or mushrooms) guides their search. However, when people explore collaboratively, their direction of search could be increasingly biased towards each other, constraining where one might otherwise be able to explore.

To foreshadow our results, our model successfully captures the difference in memory performance between the nominal and collaborative conditions—the collaborative inhibition effect—across different group sizes from 2 to 16. Our model thus provides a context-based account as to why individuals in the collaborative condition recall fewer items than in the nominal condition. Our model also captures additional recall patterns such as recency effects (enhanced recall of items from the end of the list^18^) and semantic clustering effects (semantically similar items are recalled successively^33^).

## Methods

In this section, we first provide an overview of the Gates et al. study of collaborative recall^22^. We then introduce our models of individual and collaborative recall which extend the Context Maintenance and Retrieval model (CMR)^4,30,31^. CMR was developed to explain behavioral patterns observed in a free recall task. As this is the same task that individuals in the nominal condition completed, we simply simulate recall in the nominal condition by using multiple CMR models simultaneously. To model collaborative recall, the study phase is the same as the nominal condition. We introduce an additional cognitive process to capture how group members are affected by each others’ recalls during the recall phase. The full model details can be found in the Supplementary Materials.

### Gates et al. (2022) study of collaborative recall

Gates et al. conducted a group recall study through Amazon Mechanical Turk^22^. We used their experiment 2 dataset (*N* = 1076) with groups ranging from size 2 to 16. Participants were assigned to either a nominal or collaborative condition. For each condition, there were 48 groups of size 2, 32 groups of 3, 24 groups of 4, and 12 groups of 8 and 16.

The experiment consisted of two phases: study and recall. During the study phase, participants individually viewed 60 uncategorized words. Each group saw a different list, and each participant’s presentation order was randomized. A 30-second long arithmetic filler task then followed. During the recall phase, in the nominal condition, participants were told to type as many words as they could recall from the list into a textbox. In the collaborative condition, participants within a group were placed in a chatroom together and took turns recalling. Recall proceeded in “rounds” during which each participant, in a randomized order, was given five seconds to recall a word from the list by typing it into the chatroom. If a participant recalled a not-yet-submitted word, a computerized voice read it aloud to other participants; otherwise, participants could continue attempting to recall a new list item, wait for time to elapse, select a “pass” option, or select a “I can’t recall anymore” option (which ended their participation). There was no time limit for either condition.

### Our proposed model: extending CMR to collaborative recall

**Figure 2:**
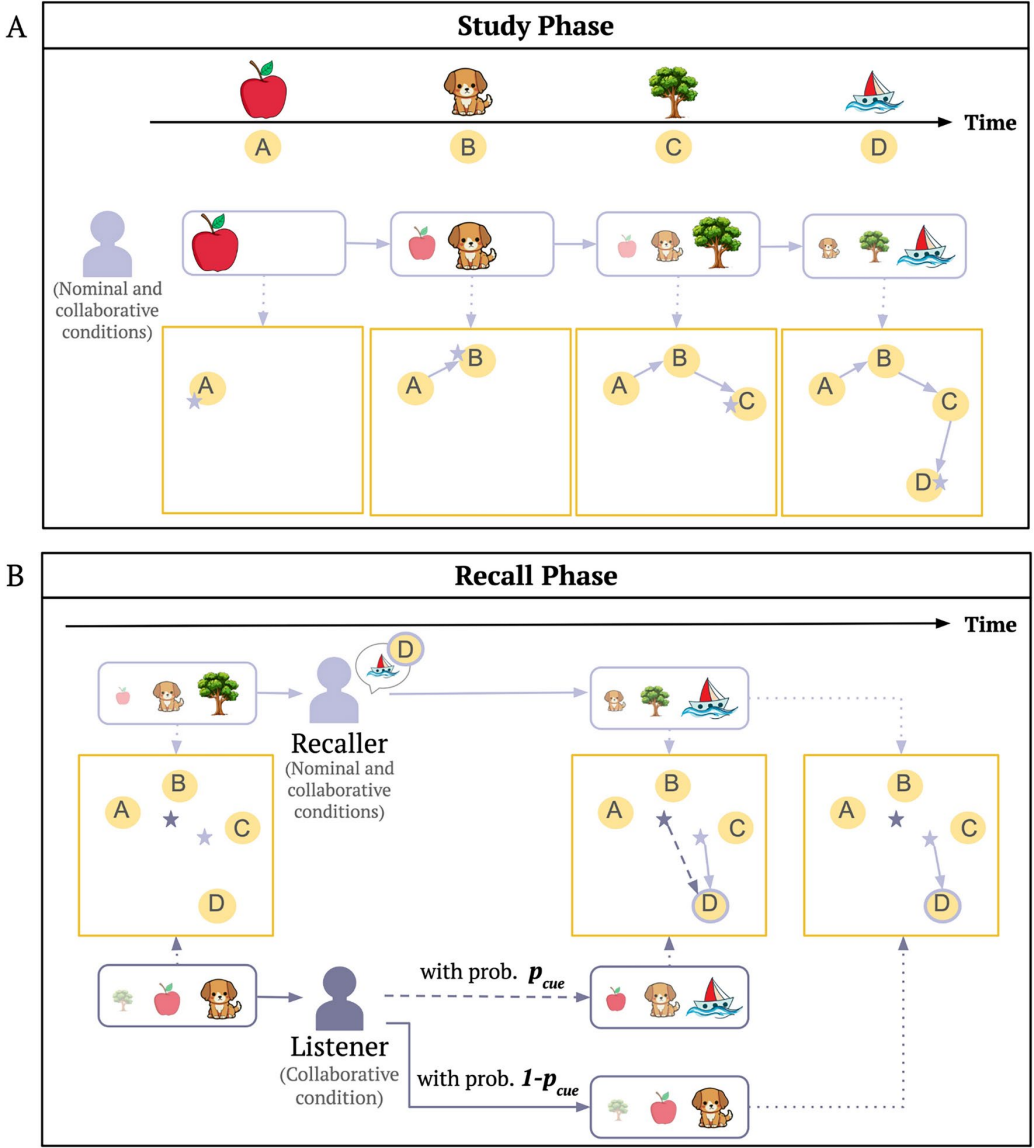
Model illustrations of individual and collaborative memory search. (**A**) Study phase for individuals in both the nominal and collaborative conditions in which items are encoded into a context space. (**B**) A snapshot of the recall phase. Individuals in the nominal condition can only be a *recaller*, and only their own recalls guide context change. Individuals in the collaborative condition can be both a *recaller* and a *listener*. As a *listener*, collaborative individuals have the chance to attend to items recalled by others (updating their context state in the same way as if one recalled the item oneself) governed by the probability $p_{cue}$.

According to the CMR model, one’s current context drifts slowly toward each new experience during the study phase and is used to drive recalls during the recall phase. To illustrate this, Fig. 2 provides an example of a free recall task with a short study list of ‘apple’, ‘dog’, ‘tree’, and ‘boat’ (also labeled as ‘A’, ‘B’, ‘C’, ‘D’ respectively).

During the study phase, when an individual studies the first item ‘apple’, it becomes associated with the part of the context space that represents the current context state (indicated by a star in Fig. 2A) of that individual. The individual’s mental context is then updated with the semantic representation of ‘apple’. When the next item ‘dog’ is presented, it becomes associated with the current context state (consisting only of the item ‘apple’ so far). The current context then drifts toward the semantic representation of ‘dog’, so that one’s current context then consists of both the newly added context of ‘dog’ as well as a faded representation of ‘apple’ (Fig. 2A). In this way, items presented nearby in time and items that share similar semantics are encoded close by in the context space. This study phase is the same across individuals in the nominal condition and the collaborative condition in Gates et al.^22^, and is simulated for each individual with a CMR model.

During the recall phase, an individual searches the context space in which the items were encoded. In a snapshot of the recall phase (illustrated in Fig. 2B), the recaller’s current context (indicated by the lilac star) is composed of the representation of item ‘tree’ as well as faded representations of ‘dog’ and ‘apple’. Which of the remaining items is retrieved depends on how close the current context state is to their study contexts. If the next recall is ‘boat’ (item D), the representation of ‘boat’ is used to update the current context state (similarly to how a studied item is used to update the current context state). The updated context is then used to drive the next recall. This process of recalling an item close to the current context and updating the current context with the recalled item repeats until one cannot recall anymore (specified by a stopping rule in the model). In the nominal condition, individuals only attend to their own recalls and thus only take on the role of recaller, which can be simulated by the CMR model. In the collaborative condition, an individual can be both a recaller and a listener who attends to the recalls of others. When it is one’s turn to be the recaller, the process is the same as described in the nominal condition and can be simulated by a CMR model. All other group members take on the role of the listener, which requires modifications to the CMR model. As a listener, there are two options. Under the probability $p_{cue}$, one listens to ‘boat’ (item D) generated by the recaller and updates their context in the same way as if they recall the item ‘boat’ on their own (see that the purple star moves to D). Under the probability $1-p_{cue}$, one ignores the item ‘boat’ generated by the recaller and their current context (indicated by the purple star) stays unchanged. Thus, in our model, not only can one use the context induced by their previous recall as an internal retrieval cue, but one can also attend to another individual’s recalled item and use it as an external retrieval cue to update their context state.

Critical to our modeling approach, we assume that the model of collaborative recall inherits the same parameter values from individual recall (which we obtain from fitting our model to participants in the nominal condition). To account for memory behavior in the collaborative condition, we only have one free parameter, $p_{cue}$, which describes the probability of listening to others’ recalls. This way, it is guaranteed that any differences we observe between the nominal condition and the collaborative condition are not a result of differences in how members in the two conditions encode and recall information from their mental context, but from the way contexts interact with each other in a group. We assume that the fundamental memory processes of how one searches their memories remain the same across individuals for whichever condition they are in.

## Results

### Nominal condition recall behavior

To simulate the experiment in Gates et al.^22^ with our proposed model, we first fit our model parameters to the free recall behavior in the nominal condition. We used Bayesian optimization^34^ to search the space of parameters and to minimize the normalized root-mean-square error (nRMSE) between model simulations and the nominal condition data across three sets of behavioral patterns: (1) how well on average words are retrieved for each position in the study list (serial position curve^18^), (2) which position in the study list recall is initiated from (probability of the first recall^18^), (3) how likely it is to recall semantically similar words at adjacent (lag 1) versus far-apart recall positions (lags 2, 3, 4). Semantic similarity is computed by finding the average cosine similarity between every pair of recalled items at different lags for their output positions^10^. Model parameters and full details of the parameter estimation procedure can be found in the Supplementary Materials.

**Figure 3:**
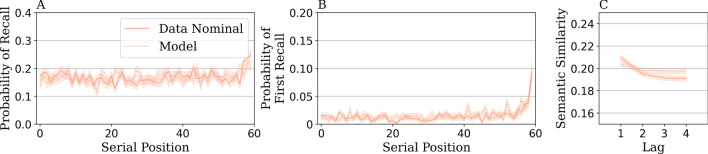
Behavioral recall patterns for individuals in the *nominal condition* and the model fit. These patterns are (**A**) the serial position curve, (**B**) the probability of first recall, and (**C**) semantic similarity by lag. The shaded error represents the standard error of the mean.

Figure 3 compares the observed patterns in the data and the model (nRMSE = -6.80). The serial position curve (Fig. 3A) and probability of first recall (Fig. 3B) in the nominal condition displayed recency effects—enhanced recall of items from the end of the list^18^. The model captured recency effects because one’s context at the start of recall is most similar to the contexts of the last few studied items. There are also semantic clustering effects in the nominal condition (Fig. 3C), as we observed higher semantic similarity at lag one than at lag four in both the data ($t(8803) = 5.59$, $p <.001$) and the model ($t(49227) = 6.07$ $p <.001$). The model captured semantic clustering effects because retrieving an item ‘apple’ updates the current context towards that item, making it easier to retrieve a semantically related item, like ‘pear’. Note that some typical free recall behaviors were not observed in the data, these being primacy effects (enhanced recall of items from the start of the list^18^) as well as temporal contiguity effects (items studied in nearby serial positions are recalled successively^11^, see further analyses in the Supplementary Materials). We think that the long list length of 60, compared with typical shorter list lengths in the range of 10 to 24 in free recall experiments^18,35,36^, contributes to the lack of these patterns (see also Hong et al.^37^). For the rest of the analyses, we focus on modeling behavioral patterns of the observed recency effects, semantic clustering effects, and memory performance.

### Collaborative condition recall behavior

**Figure 4:**
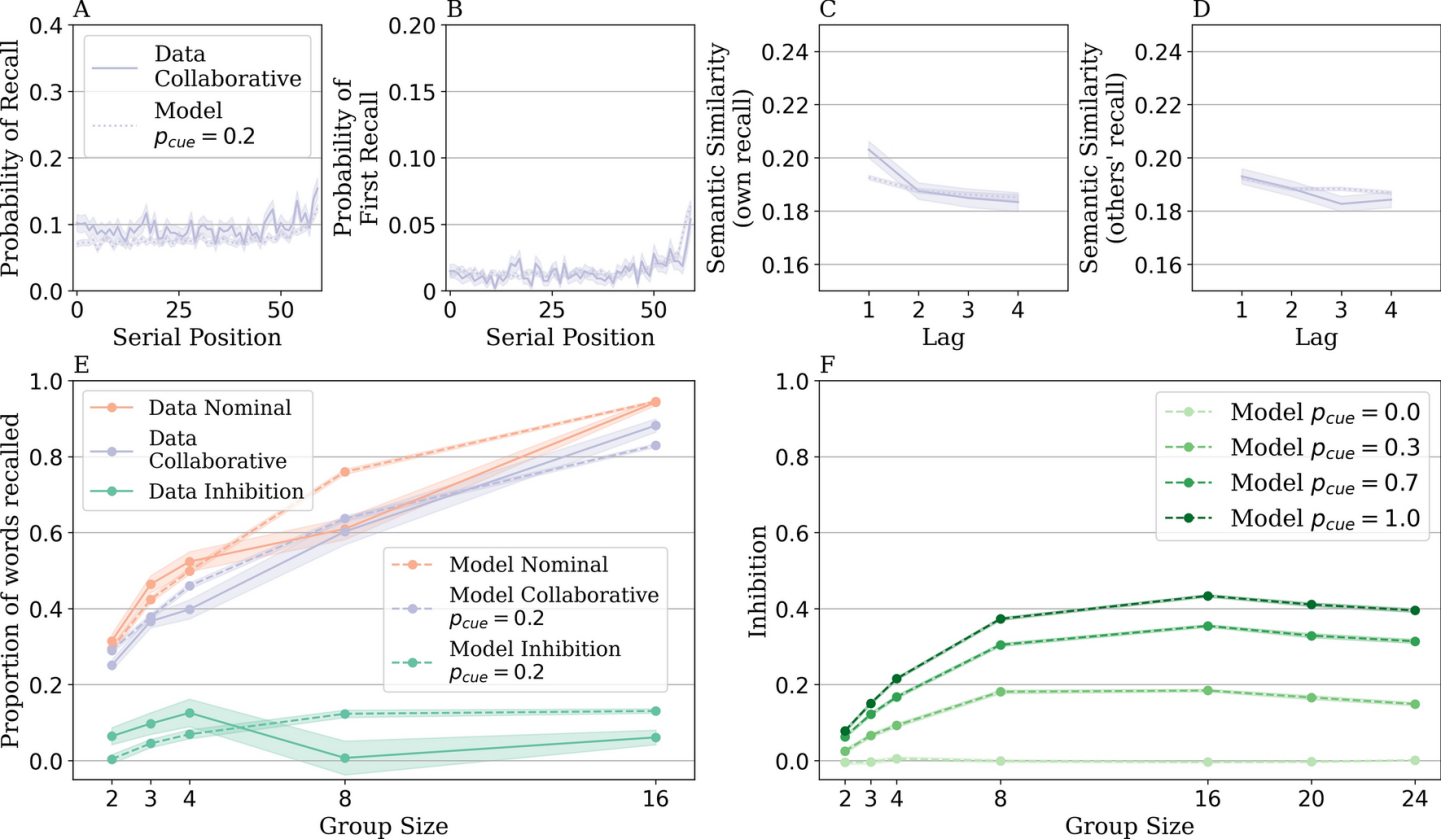
Behavioral recall patterns and performance for individuals in the *collaborative condition* and the model predictions. These patterns are (**A**) the serial position curve, (**B**) the probability of first recall, (**C,D**) semantic similarity by lag related to previous recalls of the same individual versus the entire group, and (**E,F**) the effect of group size on collaborative inhibition (calculated as the difference in the proportion of words recalled between the nominal condition and the collaborative condition) in participant data versus in the model. The model, with CMR parameters inherited from the nominal condition, best captured the collaborative data patterns with its one free parameter fit to $p_{cue} = 0.2$. The model captured the qualitative trend of collaborative inhibition by group size (which increased and then decreased as group size grew) under different values of $p_{cue} > 0$. The shaded error represents the standard error of the mean.

Our model could capture the recall behavior of individuals in the nominal condition. We next tested if the same set of parameters could also capture the recall behavior of individuals in the collaborative condition. The collaborative condition inherited its parameter set from the nominal condition as we hypothesized that the fundamental memory search processes (i.e., how context is used to encode and later retrieve items from the context space) are the same in nominal and collaborative conditions. The collaborative condition additionally has one parameter, $p_{cue}$, describing the probability of listening to others’ recall. We searched for a value from zero to one in 0.1 increments and found that $p_{cue} = 0.2$ minimized the normalized root-mean-square error between our model simulations and the collaborative condition data (nRMSE = -69.6). The fitting was done across the same behavioral patterns in the nominal condition (Fig. 4A–C) as well as one additional pattern (Fig. 4D) that characterizes the process of listening to the recalls of others. While Fig. 4C illustrates how a recall is semantically related to previous recalls of the same individual (as participants in the collaborative condition always attend to their own recalls), Fig. 4D illustrates how a recall is semantically related to previous recalls of the entire group (as participants may also attend to others’ recalls).

Participants in the collaborative condition showed typical free recall behaviors similar to that of the nominal condition, demonstrating recency effects (Fig. 4A, B) and semantic clustering effects (Fig. 4C). Participants not only retrieved items that were semantically related to their own recall (Fig. 4C), with higher semantic similarity at lag one than at lag four (data: $t(4235) = 4.01$, $p <.001$; model: $t(17307) = 3.29$, $p <.001$), but they also retrieved items that were semantically related to others’ recalls (Fig. 4D; data: $t(5504) = 2.14$, $p =.032$; model: $t(28582) = 2.68$, $p =.007$). Our model captured these patterns with its parameters fit to the recall behavior of individuals in the nominal condition. The alignment between our model’s predicted recall patterns and the observed collaborative recall patterns supports our hypothesis that one not only uses their previous recall to drive their recall context but also listens to someone else’s recall. For analyses of additional behavioral patterns to which our model was not explicitly fit, see the Supplementary Materials.

Next, we tested the key hypothesis of the present work and examined if our context-based model can capture the collaborative inhibition effect (i.e., worse memory performance in the collaborative condition than in the nominal condition). We examined this under multiple values of $p_{cue}$ in addition to the best-fit value ($p_{cue} = 0.2$) to determine if the collaborative inhibition effect can emerge as long as there is some context interaction between individuals ($p_{cue} > 0$). Figure 4E shows the effect of group size on the amount of collaborative inhibition (i.e., the difference in the proportion of words recalled between the nominal condition and the collaborative condition) as observed in the Gates et al. data and as predicted by our model. Gates et al. found significant inhibition for group sizes 3 and 4 but not for group sizes 2, 8, and 16^22^. Although our context-based model was only fit to data from the nominal condition pooled across all group sizes, its predictions on the effect of group size and on the collaborative condition’s performance align well with the data. (Fig. 4E). Moreover, as shown in Fig. 4F, our model captured the qualitative trend of collaborative inhibition by group size (which increased and then decreased as group size grew) under different values of $p_{cue} > 0$, although the model shows the decrease beginning at group size 16 whereas this occurs at group size 8 in the participant data. In the extreme case when $p_{cue} = 0$, group members do not attend to each other’s recalls, making collaborative groups equivalent to nominal ones and resulting in no inhibition predicted by our model. These results support that the performance difference between the collaborative condition and the nominal condition arose naturally from our model’s collaborative mechanism and was not sensitive to the values of the parameter we introduced to model collaborative recall. In fact, the collaborative inhibition effect shows up without fitting any parameters to the collaborative condition ($p_{cue}$ is varied at different values, with the rest of the parameters inherited from the nominal condition), demonstrating that inhibition is an emergent property of the context interaction.

### Context dynamics underlying collaborative inhibition

**Figure 5:**
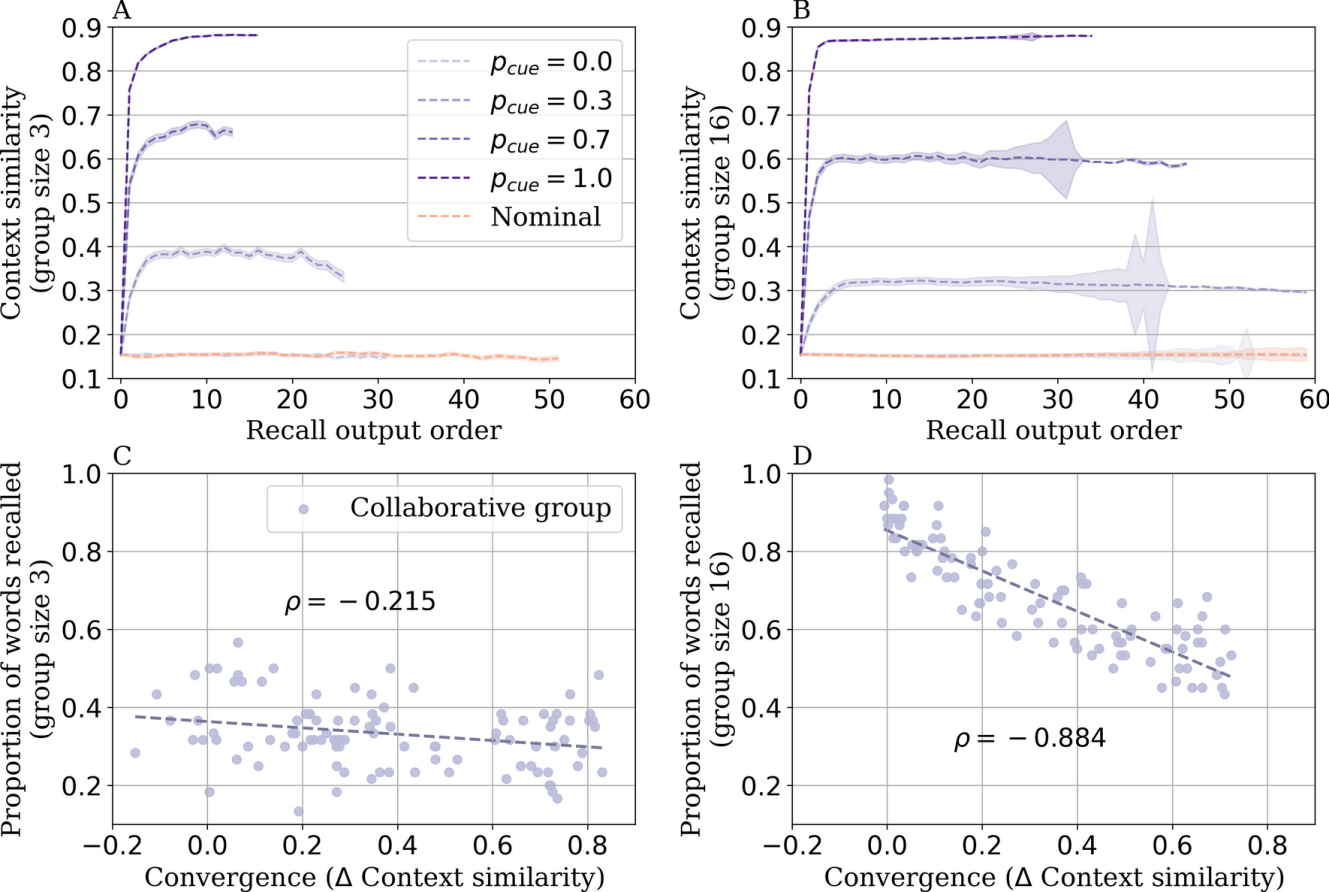
Model mechanism underlying the collaborative inhibition effect. Change in context similarity of group members throughout recall in the nominal condition and the collaborative condition for (**A**) group size 3 and (**B**) group size 16. Context convergence (defined as the difference in context similarity from early to late recalls—recall output range 0 to 10) negatively correlated with the proportion of words recalled for (**C**) group size 3 and (**D**) group size 16. All simulations were conducted with parameters fit to individuals in the nominal condition. The shaded error represents the standard error of the mean.

Our model captured the collaborative inhibition effect, where recall performance is worse in the collaborative condition than in the nominal condition. Why and how does the collaborative inhibition effect arise in the model? We hypothesized that when individuals interact in a collaborative setting, they listen to each other’s recall, and their mental contexts become similar and synchronized over time. Compared with the nominal condition where individuals use diverse and unique contexts, synchronized contexts in the collaborative condition may constrain one’s ability to recall. To evaluate if listening more to others’ recall (increasing $p_{cue}$) gives rise to more synchronized contexts in a group, we quantified the context similarity in a group by computing the average cosine similarity between context vectors of all possible pairs of simulated members per group after each recall. Figure 5A, B plot the context similarity across recall outputs for both nominal and collaborative conditions for group sizes 3 and 16. Consistent with our hypothesis that collaborative recall increases context similarity across participants, our model simulations showed that participants in the collaborative condition increased context similarity more quickly and maintained greater context similarity than in the nominal condition. To formally test this effect, we performed a two-way ANOVA to examine if the change in context similarity from early to late recalls (0, 10) is different across nominal and collaborative ($p_{cue} = 0.3$) conditions. There was a significant interaction between recall condition and recall output for group size 3 ($F(1, 396) = 193.7, p <.001$) and group size 16 ($F(1, 396) = 1052.5, p <.001$). Similarly, we also observed greater context similarity with larger values of $p_{cue}$, as there was a significant interaction between different collaborative conditions ($p_{cue} = 0.3$, $p_{cue} = 0.6$, $p_{cue} = 1.0$) and recall output for group size 3 ($F(1, 1196) = 585.0, p <.001$) and group size 16 ($F(1, 1196) = 3178.8, p <.001$). When $p_{cue} = 0$, context similarity in nominal and collaborative conditions was similar as collaborative group members were no longer affected by others’ recalls. These results support that listening to each others’ recalls in a collaborative setting guides individuals to reach more similar areas of the memory space, giving rise to more synchronized mental contexts.

We next tested if greater context similarity in a collaborative group relates to their ability to search the memory space, i.e., memory performance. To explore this, we simulated collaborative groups with different amounts of context convergence (by uniformly sampling $p_{cue}$ values from 0 to 1), measured as the change in context similarity from early to late recalls (0 to 10). Consistent with our hypothesis, we found worse memory performance in groups with higher context convergence (Fig. 5C, D), with negative Spearman’s correlations between the change in context similarity and the proportion of words recalled by the group ($\rho (99) = -.215, p =.030$ for group size 3; $\rho (99) = -.884, p <.001$ for group size 16). These results support that a context-based account can explain the behavior observed in the collaborative condition: collaborative group members are influenced by the context of others, which constrains their ability to search their memories in the context space.

### Context dynamics underlying group size effect

**Figure 6:**
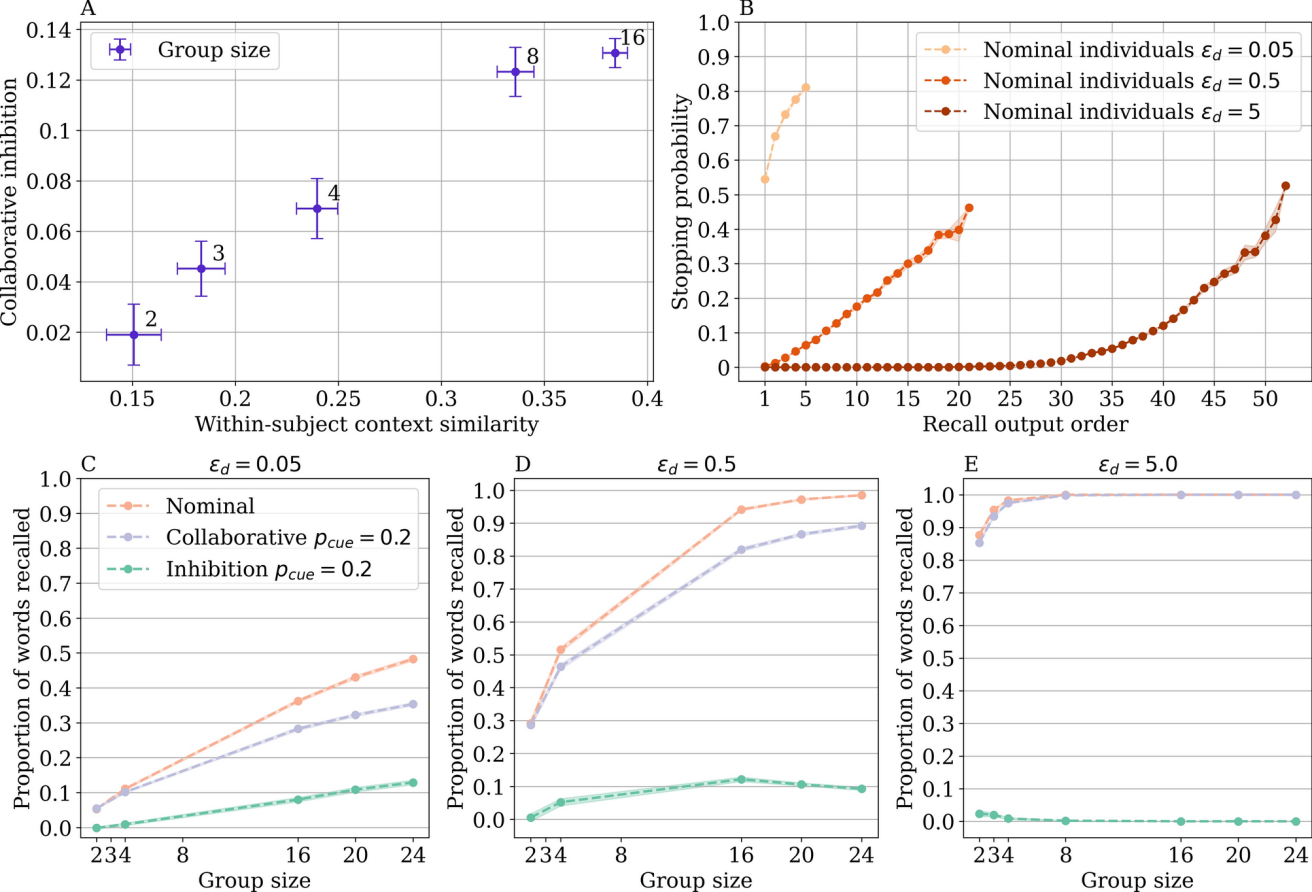
Model mechanisms underlying the group size effect. (**A**) Within-subject context similarity averaged by group size (calculated as the similarity between each individual’s start-of-recall context and their context after ten items were recalled) increased with inhibition averaged by group size. (**B**) The probability of stopping recall by output position. At smaller values of the parameter $\epsilon _d$, the model is more likely to terminate recall, and therefore, retrieves fewer items. (**C**) With a high stopping probability ($\epsilon _d = 0.05$), none of the nominal group sizes reached ceiling performance (i.e., recalling nearly the entire list), and inhibition increased with group size. (**D**) With a smaller stopping probability ($\epsilon _d = 0.5$), inhibition began to decrease when the nominal condition reached ceiling performance at group size 16. (**E**) With an even smaller stopping probability ($\epsilon _d = 5.0$), the nominal condition reached ceiling performance at small group sizes, and inhibition largely disappeared. All simulations used the best-fit parameter $p_{cue} = 0.2$, with the remaining CMR parameters inherited from the nominal condition. Error bars and shaded errors represent the standard error of the mean.

Our model captured not only the collaborative inhibition effect but also how the amount of inhibition first increases and then decreases by group size. Why and how does the group size effect arise in the model? The first effect we analyzed was the initial increase in inhibition by group size in the model. We have shown in Fig. 5 how collaborative inhibition was associated with more constrained memory search, as mental contexts were more synchronized across individuals in the collaborative condition than in the nominal condition. We think that the same mechanism can account for the initial increase of collaborative inhibition by group size—collaborative group sizes that experience more inhibition have individuals whose search is more constrained in the memory space. Instead of using context similarity across all pairs of members in a group (which is confounded by group size), we used a within-subject metric to compute the context similarity between each individual’s start-of-recall context with their context after ten items had been recalled (instead of the end-of-recall context to not be confounded by recall performance). This similarity metric approximated how much of the context space one traversed during recall—the more the similarity, the less area searched. We found that in groups sizes 2 to 16, when inhibition increased with group size, the within-subject context similarity after ten recalled items also increased (Fig. 6A), and this effect was significant ($p =.006$) under a permutation test where we randomly shuffled the labels for 2, 3, 4, 8, and 16 group sizes 1000 times. These results support our context-based account of collaborative inhibition when group size first increases. One’s memory search becomes more constrained and reaches fewer areas in the context space when listening to others’ recall, and this effect is stronger when there are more individuals in the group.

The second effect we analyzed was the eventual decrease in collaborative inhibition when the group size was large. We hypothesized that the decreasing trend arises from the nominal condition reaching ceiling performance in our model. Starting at a certain group size, the nominal condition is able to recall nearly all items on the list. Larger nominal groups thus have little room to recall more items to improve performance. At the same time, the collaborative condition recalls successively more items as group size increases. Both these trends could contribute to the performance difference between the two conditions reducing once the nominal condition reaches ceiling performance. To test this, we varied the stopping parameter in our model, $\epsilon _d$, to control when the nominal condition hits ceiling performance. A smaller $\epsilon _d$ leads to a larger stopping probability and decreases the recall amount, whereas a larger $\epsilon _d$ increases the recall amount (Fig. 6B). If the ceiling effect contributed to the decreasing trend in inhibition, then removing the ceiling effect should remove the decreasing trend. Indeed, when nominal groups recalled fewer items and were not influenced by a ceiling effect (with a small value of $\epsilon _d$ and a large stopping probability), our model predicted increasing inhibition with group size (Fig. 6C). With a larger value of $\epsilon _d$ and a smaller stopping probability, inhibition decreased once the nominal condition hit ceiling performance (Fig. 6D). When the stopping probability was very low (with a very large value of $\epsilon _d$), ceiling performance was reached in even smaller groups in the nominal condition, and inhibition largely disappeared as both groups were able to recall the majority, if not all, of the list (Fig. 6E). These two mechanisms together can explain how the model qualitatively predicted the trend of an initial increase in inhibition followed by a subsequent decrease as group size grew.

## Discussion

Context has been able to explain many memory phenomena in individuals^4,6–12,18^. We proposed that a context-based account would also be able to explain how groups recall information together: in collaborative recall, the context of an individual not only evolves on its own but is also influenced by the context of others in the group. To test our account, we built a computational model of collaborative recall in groups, extended from the Context Maintenance and Retrieval (CMR) model which captures how individuals recall information in isolation^31^. By comparing our model’s simulations to an empirical dataset of nominal and collaborative group recall^22^, we found that the model can capture the collaborative inhibition effect across different group sizes as well as recency effects and semantic clustering effects. We also found that the contexts of individuals in the collaborative condition converged in the context space more than in the nominal condition, and this convergence was negatively associated with recall performance. These results support our proposed account of collaborative inhibition: Minds within a collaborative group become aligned or synchronized with each other, thus missing opportunities to recall unique information that others may not have considered. We now turn to the broader implications.

Multiple accounts have been proposed previously to explain why the collaborative condition recalls less information than the nominal condition. For one, the account of *retrieval disruption* theorizes that each individual has an idiosyncratic retrieval strategy that is disrupted when listening to others’ recalls, giving rise to non-optimal recalls^24,27^. Alternatively, the *retrieval inhibition* account argues that an additional mechanism operates whereby when a group member recalls an item, as-yet-retrieved items are suppressed in listeners’ memory, making the remaining items less likely to be retrieved^28^. Last, the account of *limited exploration* has been proposed in which members of a collaborative group constrain other members’ exploration of memory, as they found that collaborative dyads—consisting of two members recalling together—reached fewer categories than nominal dyads when recalling categorized lists^29^.

Our context-based account helps unify these previous accounts in a computational model of collaborative recall. Computational models have the advantage over verbal or descriptive theories to precisely characterize a hypothesized cognitive process. Our proposed model for collaborative recall details how participants’ contexts evolve when items are encoded and how they interact with each other during recall. Our computational account based on context is different from other verbal accounts of collaborative inhibition. For example, although the retrieval disruption account—where individuals’ idiosyncratic retrieval strategy is disrupted by others’ recalls—can intuitively explain the observed inhibition effect, it is left unspecified what an individual’s ‘idiosyncratic retrieval strategy’ is and why it is ‘disrupted’ when listening to others’ recalls. In comparison, we mathematically formulate where the disruption comes from (as the listener’s context is disrupted by and becomes similar to the recaller’s context) and demonstrate its effect on the downstream memory performance. Similarly, our context-based model also captured the idea of limited exploration. While previous empirical findings have supported this account (as two members recalling together reached fewer categories than recalling separately^29^), our computational model demonstrated that such convergence of memory—in a context space—is an emergent property when individuals in a collaborative condition interact with each other. Additionally, we verified its relationship with recall performance, as the amount of convergence is negatively associated with recall performance. Finally, the major advantage of our context-based account over the retrieval inhibition account lies in its parsimony. Our computational model captured the performance difference between the nominal condition and the collaborative condition without introducing an inhibitory mechanism, where as-yet-retrieved items are suppressed in listeners’ memory.

Despite the advantages, there are also challenges associated with building computational models. This is especially true when the number of parameters is large, as “designing independent tests of the model may be difficult since its ten parameters and numerous countervailing processes make unambiguous predictions hard to come by”^38^. In other words, there is risk in building a complicated model (with a large set of parameters) to capture a simple set of behavioral patterns (such as memory performance in the nominal condition and the collaborative condition), as one can always introduce new processes or alter parameter values during post hoc theorizing.

Our modeling circumvented these issues despite its complexity. First of all, for the model of collaborative recall, except for the one process that describes the interaction between contexts, all other model components are inherited directly from the CMR model that captures individual recall. The complexity of the CMR model has been well justified, given its success in capturing a wide range of behavioral patterns in free recall literature^4,30–32^. Second, when we examine how the collaborative condition differs from the nominal condition, there is only one free parameter ($p_{cue}$), as the rest of the parameters are fixed values obtained from the nominal group. If we let a large number of parameters vary between the two conditions, it would be trivial to account for their performance differences, as one can easily find a set of parameter values to fit the data performance perfectly, when the data patterns are simple and the number of parameters is large. Lastly, with one free parameter, we carried out an analysis on model flexibility^39,40^, where inhibition arises under all positive values of $p_{cue}$, demonstrating that inhibition is an emergent property of the context interaction.

There are two existing computational models of collaborative recall, namely an agent-based model (ABM)^22,41^ and an extension of the Search of Associative Memory (SAM) model^42,43^. The agent-based model can account for the collaborative inhibition effect by introducing an inhibition mechanism: when an individual recalls an item, it reduces activations of other items based on their similarity to that recalled item for every individual in the group^41^. The SAM model posits that group members listen to the recall of whoever recalls first, and this item is then used to cue their next recall^43^. While both the ABM and the SAM model have successfully captured the collaborative inhibition effect, to account for empirical data, they require fitting model parameters simultaneously to the nominal and collaborative average recall amounts^22,43,44^. In contrast, our context-based model takes a novel approach and demonstrates that inhibition is an emergent property of context interaction: using the same parameter set that was fit only to the nominal condition, the model was able to predict the collaborative inhibition effect and recall behaviors in the collaborative condition. Nevertheless, to compare our model with the ABM and the SAM model directly, we conducted additional analyses with all three models fit to the nominal condition to predict the collaborative inhibition effect (see Supplementary Materials). Results showed that our context-based model captured the serial position curve of the collaborative condition more accurately than the ABM or the SAM model, particularly for early list positions. Regarding collaborative inhibition, ABM over-predicted inhibition, especially for large group sizes. The SAM model predicted a similar amount of inhibition by group size as the context-based model. We do not find it surprising that SAM has a similar ability as our context-based model in predicting the collaborative inhibition effect. Both SAM and CMR (from which our model was extended) have a history of successfully accounting for various behavioral patterns in memory search^31,42^. However, CMR is a sparser model as episodic memory is retrieved only from item-context associations, whereas the SAM model requires retrieval from both item-context associations and item-item associations; additionally, SAM assumes two separate memory stores: a short-term store and a long-term store, whereas CMR does not require such a distinction. We believe that a model’s value lies not only in its ability to account for data patterns but also in how well it can be analyzed to enhance our understanding of underlying mechanisms. The sparsity of our context-based account allows us to capture the collaborative inhibition effect while simultaneously revealing where this effect comes from in the model. Our model results demonstrated that when minds within a collaborative group become too convergent in the context space (with high context similarity), they miss opportunities to recall unique information that others might not have considered. This insight provides a clear understanding of the mechanism driving the collaborative inhibition effect.

Our proposed context-based model could be extended in the future to account for a related paradigm in external cuing literature, namely part-set cuing^45^, given the similarity between a part-set cuing paradigm and a collaborative recall paradigm. In collaborative recall, external retrieval cues are provided by the recalls of other group members. In part-set cuing, individuals complete a free recall task in which, after studying a list of items, some participants are provided a subset of list items by the experimenters as retrieval cues. These cued participants recall fewer non-cue items than participants who did not receive any cues (for reviews, see Bauml and Nickerson^46,47^). Part-set cuing has been explained by similar accounts as collaborative inhibition, specifically retrieval disruption^48^ and retrieval inhibition^49,50^. However, context may also account for the reduced recall performance observed in cued individuals. Recent part-set cuing research has posited that cues can reactivate the original study context after access to it was impaired^51,52^. Following our context-based account, an item (whether externally provided by the experimenter, by another participant or internally by oneself) updates the location of one’s current context to be more like the item. Cues may disrupt one’s context, making it more similar to the context of cue items than non-cue items. This, in turn, may limit one’s ability to recall non-cue items. This would provide a more parsimonious account by not assuming additional mechanisms such as inhibition (for a related context-based account for the effect of single, experimenter-provided cues, see Cornell et al.^10^).

Our computational modeling work offers unique contributions to collaborative memory research. We provide a strong test of our proposed context-based account by demonstrating its ability to capture key recall patterns in collaborative recall without directly fitting parameters to the data in the collaborative condition. Our model of collaborative recall can capture the collaborative inhibition effect, as listening to others recall constrains where one could otherwise be able to search in the context space. Moreover, our model can account for detailed patterns of collaborative recall behavior, including the recency effects and the semantic clustering effects. Taken together, our results support the important role of context in explaining a range of memory findings across individuals and groups.

## Supplementary Information

Supplementary Information.

## Data Availability

The analysis code is publicly accessible here: https://osf.io/r6xaj. The dataset used in the current study is from Gates et al.^22^, which is publicly available here: https://osf.io/txe9a.
