# Supplementary material for: A context-based model of collaborative inhibition during memory search
Source: Sci Rep. 2024 Nov 12;14:27645. doi: 10.1038/s41598-024-78517-w (PMC11558009; doi:10.1038/s41598-024-78517-w)
Supplement: Supplementary file 1 — Supplementary Information. [file 41598_2024_78517_MOESM1_ESM.pdf]

Supplementary Materials

Our Proposed Model

Study Phase (nominal and collaborative conditions) During the study phase, participants in nominal and collaborative conditions individually studied 60 words. To model this, we follow the exact processes of CMR and assume that simulated members of both nominal and collaborative groups encode a list of items in the same way. When an individual studies an item from the list, their current context drifts towards the memory representations of the recently encountered item. The state of the context at time t is given by:

$$c_t = \rho c_{t-1} + \beta_{enc} c^{IN} \tag{1}$$

where c^{IN} is the retrieved context induced by the encountered item, parameter $\beta_{enc} \in [0,1]$ determines the rate at which context drifts toward that presented item's context c^{IN}, and ρ is a normalizing scalar that renders $\| c_t \| = 1$. The presented list item activates its pre-experimental context c^{IN}:

$$c^{IN} = M^{FC}_{pre} f_t \tag{2}$$

where M^{FC}_{pre} stores item-to-context associations that existed before the experiment, and f_t is a binary vector that is all zeros except at the presented item's position. Therefore, $M^{FC}_{pre} f_t$ is the context previously associated with the presented item. In addition to these fixed pre-experimental item-to-context associations held in M^{FC}_{pre}, there are also experimental item-to-context and context-to-item associations held in M^{FC}_{exp} and M^{CF}_{exp} that capture new learning in the experiment. These matrices are initialized to zero and are updated during the study phase. Specifically, when an item is presented, a new association is formed via the Hebbian outer-product learning rule:

$$\Delta M^{FC}_{exp} = \Delta M^{CF}_{exp} = f_t c^T_{t-1} \tag{3}$$

The overall effect of having context drift slowly towards each newly presented item, together with the process of associative learning, is that items presented nearby in the study list tend to be associated with similar context states.

Recall Phase (nominal condition) During the recall phase, participants in the nominal condition recalled items separately while participants in the collaborative condition recalled items together. The recall process for individuals in the nominal condition follows the same recall process as the CMR model. Specifically, during recall, the current context $c_{t-1,j}$ of a simulated participant j drifts towards the retrieved context of the just recalled item c^{IN}_{rec}:

$$c_{t,j} = \rho c_{t-1,j} + \beta_{rec} c^{IN}_{rec} \tag{4}$$

Here, context continues to drift during recall following the same process during study (Equation 1), but at a different rate as determined by $\beta_{rec} \in [0,1]$ and with c^{IN} expressed differently. During study, an item only retrieves its pre-experimental context when it is presented; however, when an individual recalls an item, its retrieved context activates both its pre-experimental context ($M^{FC}_{pre} f_t$) and its experimental context formed during study ($M^{FC}_{exp} f_t$). The extent of retrieving an item's pre-experimental versus experimental context is determined by a parameter, $\gamma_{fc} \in [0,1]$, such that:

$$c^{IN}_{rec} = (1-\gamma_{fc}) M^{FC}_{pre} f_t + \gamma_{fc} M^{FC}_{exp} f_t \tag{5}$$

Once context drifts towards this retrieved context, which items are likely to be recalled? The support (or activation) a^{IN} at time t for recalling different items depends on both how much the current context c_t matches with items' experimental contexts (stored in M^{CF}_{exp}) as well as items' pre-experimental contexts (stored in M^{CF}_{pre}; see description below). The relative activation of these associations is determined by a parameter, $\gamma_{cf} \in [0,1]$, such that:

$$a^{IN} = \gamma_{cf} \phi_i M^{CF}_{exp} c_t + (1-\gamma_{cf}) M^{CF}_{pre} c_t \tag{6}$$

Here, M^{CF}_{pre} is a matrix representing pre-experimental context-to-item associations. To capture semantic clustering effects observed at recall, M^{CF}_{pre} begins as an identity matrix and each element in M^{CF}_{pre}, with indices m and n, is additionally incremented by a semantic association between items m and n, determined by taking the cosine similarity of the two items' GloVe model embeddings[1]. Each entry of the semantic association is additionally raised to the power of λ before being scaled by the constant s_{cf} to match with human semantic representations.

To simulate which item to recall next based on items' support in a^{IN}, the model also needs a retrieval rule and a stopping rule. We use the softmax function as the retrieval rule, $p_i = e^{k a^{IN}_i} / \sum_j e^{k a^{IN}_j}$, where a^{IN}_i is the support to retrieve item i and

the parameter k determines the amount of noise. Once an item is retrieved, the context state drifts again, towards that item's retrieved context following Equation (5); cuing with this updated context state supports the retrieval of new items. These retrieval and context updating processes continue until what is determined by a stopping rule: the probability of stopping at each time point is expressed as $p_{stop} = e^{-\varepsilon_d a_{nr}^{IN}/a_r^{IN}}$, where a_r^{IN} indicates the summed support for already-recalled items, a_{nr}^{IN} indicates the summed support for not-yet-recalled items, and ε_d is a scaling factor[2–4]. Overall, because items studied nearby in the list are tied to similar context states during encoding, subsequent recalls are likely to be nearby items on the list; they are additionally likely to be items semantically similar to the current context.

Recall Phase (collaborative condition) While individuals in the nominal condition update their context using only their own recalls, individuals in the collaborative condition additionally can listen to recalls of other members in their group. In Gates et al., collaborative recall proceed in "rounds" in which each group member was randomly selected and given a chance to recall[5]. Thus for every recall round in our model, we randomly selected a simulated participant to recall an item. When a simulated participant j recalls an item in our model, their own context drifts towards that item's retrieved context following Equation (5). Because everyone else in the chatroom could hear this recalled item, we let all other simulated participants $i \neq j$ have the chance to use this item as a retrieval cue under probability $p_{cue} \in [0,1]$ by drifting their internal context $c_{t-1,i}$ towards the cue's retrieved context $c_{cue,i}$. Under probability $1 - p_{cue}$, participants ignored this item and maintained their current context, $c_{t-1,i}$:

$$c_{t,i} = \begin{cases} \rho c_{t-1,i} + \beta_{rec} c_{cue,i}, & \text{with probability } p_{cue} \\ c_{t-1,i}, & \text{with probability } 1 - p_{cue} \end{cases} \tag{7}$$

Notice that the same parameter β_{rec} governs how much one's context drifts towards someone else's recall compared with that towards one's own recall in Equation (5).

We fit CMR to the free recall behavior in the nominal condition across three sets of behavioral patterns: (1) serial position curve, (2) probability of first recall, and (3) semantic similarity probability at lags +1 to +4. We used Bayesian optimization[6] to search the space of parameters and to minimize the normalized root-mean-square error (nRMSE) between our model simulations and the nominal condition data. The behavioral patterns include points on the semantic similarity plots, as well as averages of the first 5 points, the middle 10 points, and each of the last five points from the serial position curve and the probability of first recall curve. The model parameters for the nominal condition are: $\beta_{enc} = 0.594$, $\beta_{rec} = 0.871$, $\gamma_{fc} = 0.297$, $\gamma_{cf} = 0.344$, $s_{cf} = 0.685$, $k = 2.693$, $\varepsilon_d = 0.585$, and $\lambda = 1.751$.

Additional Behavioral Patterns

In this section, we compared additional behavior patterns over the data and the model. We examined how likely it is to recall items studied consecutively in the study list as predicted by our model to those observed in the data. To do this, we plotted the conditional response probability for both conditions (computed by dividing the number of times that a transition to each lag is actually made by the number of times that it could have been made for each serial position[7]; the first four recalls were excluded to minimize the contribution from recency effects). Individuals in both nominal and collaborative conditions exhibited a lack of temporal contiguity effects (i.e., items studied in nearby serial positions are recalled successively[7]; Figure S1). Our model aligned with this behavioral pattern despite not being directly fit to these effects (Figure S1).

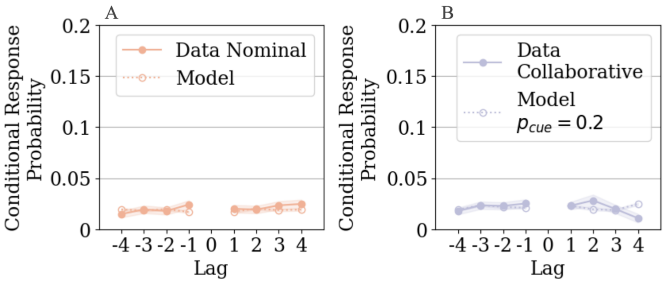

Figure S1. Conditional response probability in the data and the model in the (A) nominal and (B) collaborative conditions, computed by dividing the number of times a transition of that lag is actually made by the number of times it could have been made. Our model predicted this pattern observed in data with parameters fit over other individual recall behaviors in the nominal condition. The shaded error represents the standard error of the mean.

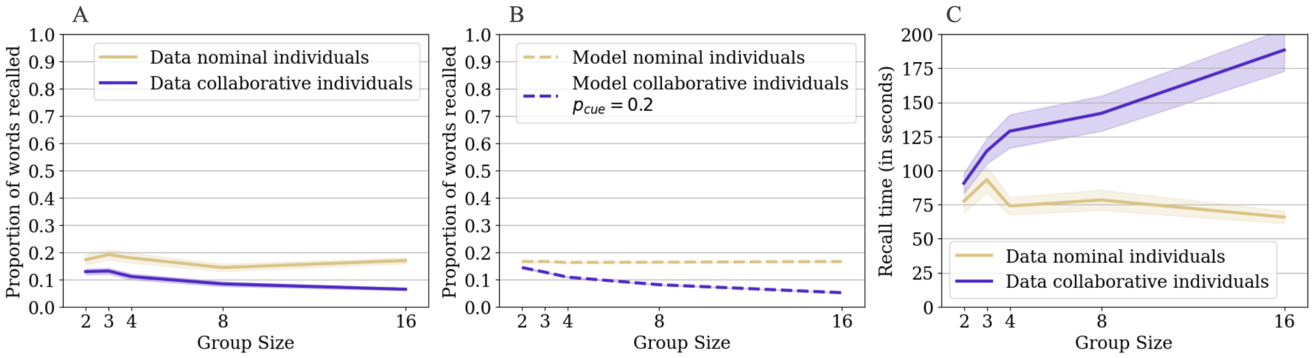

Figure S2. Individual recall performance and recall time in the data and the model. In both (A) the data and (B) the model, the individuals in the nominal condition recalled more than individuals in the collaborative condition, and this difference between the conditions increased with group size. Our model predicted these performance patterns with its parameters fit to individual recall behaviors in the nominal condition. (C) In the data, individuals in the collaborative condition spent more time recalling than individuals in the nominal condition, and this difference between conditions increased with group size. The shaded error represents the standard error of the mean.

Prior work has found that strong semantic associations between items in the study list can reduce the use of temporal organization[8–10]. However, it is unlikely to be the main factor that accounts for the lack of temporal contiguity effects, as Gates et al. used uncategorized lists in their experiment[5]. Instead, we think that a long list length of 60, compared with typical shorter list lengths in the range of 10 to 24 in free recall experiments, contributed to the lack of these patterns. Prior work has identified that as list length increases from 20 to 40, temporal organization becomes weaker[10,11] as there is increased competition among items to be recalled, creating more chances for long-distance recall transitions[10]. Indeed, we observed a weaker reliance on temporal versus semantic organization in our model fit ($\gamma_{cf} = 0.344$) compared with previously reported CMR model fits to free recall experiments (γ_{cf} are 0.895, 0.780, and 0.669; Lohnas et al.[12]). The parameter γ_{cf} controls the relative activation of temporal versus semantic associations during item retrieval.

In addition to conditional response probability, we considered the average proportion of items recalled per individual (as opposed to summing them up across all individuals and excluding duplicates which was analyzed in the main results) and found that individuals in the nominal condition recalled more items than those in the collaborative condition, and this difference increased with group size both in the data (Figure S2A) and the model (Figure S2B). Our model was able to capture this effect as we found that in our model, one's memory search becomes constrained when listening to others' recalls, and the strength of this effect increases with group size (see Figure 6A in the main text). Next, we examined the differences in recall time across group sizes. In the experiment, individuals in the collaborative condition had to wait longer before recalling as group size increased, compared with that in the nominal condition. As such, we observed that the total recall time of individuals in the collaborative condition increased with group size (Figure S2C). It is possible that this methodological difference between conditions could have influenced performance. For instance, Gates et al. mentioned that recalling with more individuals may increase competition and benefit performance. This could encourage members to try harder to recall items. Alternatively, it could lead members to adopt strategies to avoid having their own recall interrupted by others[5]. Additional work would be needed to examine such social or motivational factors. We could not model this time difference as our model only predicts which item will be recalled but not the recall time. Nonetheless, our model is still able to capture key behavioral patterns, such as collaborative inhibition, without explicitly considering recall time.

Comparing models of collaborative recall

We compared our context-based model with existing computational models of collaborative recall, namely an agent-based model (ABM)[13] and an extension of the Search of Associative Memory (SAM) model[14,15]. To simulate the empirical experiment in both models, we followed a similar fitting procedure we used with our model. Specifically, we tested how well the same parameter sets, fit to the nominal condition for each model, predicted recall behavior in the collaborative condition. We fit each model to the free recall behavior of individuals in the nominal condition by minimizing the normalized root mean squared error (nRMSE) between the data and the model across two patterns: the serial position curve and the probability of first recall. The semantic similarity by lag plot was excluded in the fitting procedure as neither model, as implemented in Luhmann & Rajaram[13] and Mannering et al.[15], could account for the effect of semantic relatedness. The best-fit model parameters for the ABM are: $\alpha = 0.773$, $\beta = 0.198$, $\gamma = 0.810$, and number of rounds = 15. The model parameters for the SAM model are:

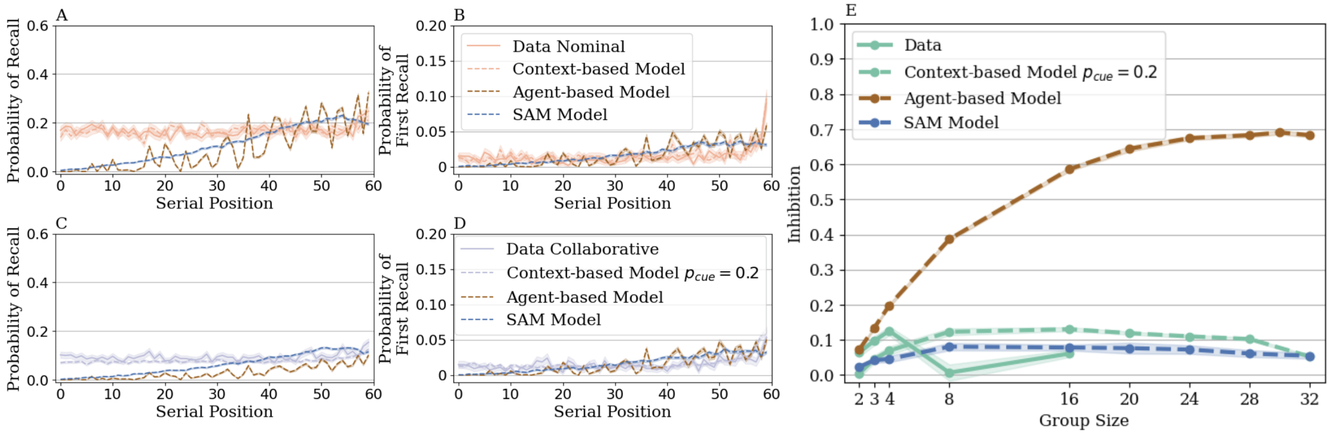

Figure S3. Recall behavioral patterns and collaborative inhibition were compared between the context-based model and two existing computational models of collaborative recall, an agent-based model (ABM) and an extension of the Search of Associative Memory (SAM) model. The ABM and the SAM model were fit to (A) the serial position curve and (B) the probability of first recall in the nominal condition, and were used to predict (C) the serial position curve, (D) the probability of first recall in the collaborative condition, and (E) the amount of collaborative inhibition by group size (calculated as the difference in the proportion of words recalled between the nominal condition and the collaborative condition). The shaded error represents the standard error of the mean.

$a = 0.03$, $b = 0.15$, $c = 0.28$, $d = 0.005$, $e = 0.67$, $f = 0.87$, $g = 0.55$, $K_{max} = 33$, and $L_{max} = 6$.

Figures S3A–B show that our context-based model captured the observed recall behavior of the nominal condition better than the other two models. The serial position curve (Figure S3A) and probability of first recall (Figure S3B) in the data displayed recency effects – enhanced recall of items from the end of the list[16]. While all three models predicted a similar probability of recall for end-of-list items, the ABM and the SAM model failed to capture the probability of recall for the earlier serial positions. In the SAM model, this may be a result of its use of cues assembled in a short-term memory store to retrieve items. At the start of recall, end-of-list items are more likely to comprise these cues, so recall tends to begin with these items. Then, because nearby items spend more time together in the short-term store, later list items, being closer to the initially recalled ones, have a higher chance at recall. While the SAM model has been seen to capture serial position effects[14], it is possible the long list length of 60 items made it difficult for earlier list items to become associated with later list items. As for the ABM, it was not developed to capture behavioral patterns like the serial position curve; it was only developed to capture recall performance. In the ABM, end-of-list items are more active, as the activations of items learned earlier in the list get reduced during encoding, contributing to the lower recall probability of early list items.

We then tested how well the same parameter sets, fit to the nominal condition for each model, predicted recall behavior in the collaborative condition. The ABM and the SAM model did not have additional parameters to be fit in the collaborative condition. In both models, when a recall is made by an individual in the collaborative condition, it is always used as a retrieval cue by other members in the group. In our context-based model, we assume that a group member does not always get cued by the recalls made by others in the group. Instead, the probability of listening to another member's recall is controlled by a parameter p_{cue}, which was set to the best fitting value $p_{cue} = 0.2$ (as detailed in the Results section "Collaborative condition recall behavior"). In the data, the collaborative condition exhibited recency effects, similar to the nominal condition, seen on both the serial position curve (Figure S3C) and the probability of first recall (Figure S3D). Our model predicted these patterns better than the other models, particularly in capturing the recall probability of earlier serial positions on the serial position curve (Figure S3C).

We then examined the collaborative inhibition effect predicted by all three models using the same parameter sets fit to the nominal condition. The SAM model predicted a similar amount of inhibition by group size as the context-based model. The ABM did the worst at predicting the amount of collaborative inhibition by group size, particularly for large groups of size 8 and 16, where it over-predicted inhibition (Figure S3E). This discrepancy is likely due to the high forgetting rate, and few recall rounds ($\beta = 0.198$ and number of rounds = 15). Gates et al., in contrast, fit the ABM directly to the performance of the nominal and collaborative conditions, and they obtained a fit with a lower forgetting rate, and more recall rounds ($\beta = 0.03$ and number of rounds = 22) that better captured performance in large groups[5].

Taken together, only the context-based model was able to account for both recall behaviors and the amount of collaborative

inhibition observed in the data, although SAM was also capable of predicting a similar amount of collaborative inhibition by group size. The novelty of our modeling approach lies in our assumption that the fundamental memory processes (characterized by model parameters) of the collaborative condition are inherited from the individuals in the nominal condition. Thus, while prior work has fit models to the performance of both the nominal and collaborative conditions directly, we demonstrated that collaborative inhibition is an emergent property.

References

1. Pennington, J., Socher, R. & Manning, C. D. Glove: Global vectors for word representation. In *Proceedings of the 2014 conference on empirical methods in natural language processing (EMNLP)*, 1532–1543 (2014).
2. Cornell, C. A., Norman, K. A., Griffiths, T. L. & Zhang, Q. Improving memory search through model-based cue selection. *Psychol. Sci.* **35**, 55–71 (2024).
3. Kragel, J. E., Morton, N. W. & Polyn, S. M. Neural activity in the medial temporal lobe reveals the fidelity of mental time travel. *J. Neurosci.* **35**, 2914–2926 (2015).
4. Zhang, Q., Griffiths, T. L. & Norman, K. A. Optimal policies for free recall. *Psychol. Rev.* **130**, 1104–1124 (2023).
5. Gates, V., Suchow, J. W. & Griffiths, T. L. Memory transmission in small groups and large networks: An empirical study. *Psychon. Bull. & Rev.* 1–8 (2022).
6. Mockus, J. The application of bayesian methods for seeking the extremum. *Towards global optimization* **2**, 117–129 (1998).
7. Kahana, M. J. Associative retrieval processes in free recall. *Mem. & Cogn.* **24**, 103–109 (1996).
8. Polyn, S. M., Erlikhman, G. & Kahana, M. J. Semantic cuing and the scale insensitivity of recency and contiguity. *J. Exp. Psychol. Learn. Mem. Cogn.* **37**, 766 (2011).
9. Healey, M. K. & Uitvlugt, M. G. The role of control processes in temporal and semantic contiguity. *Mem. & Cogn.* **47**, 719–737 (2019).
10. Hong, M. K., Gunn, J. B., Fazio, L. K. & Polyn, S. M. The modulation and elimination of temporal organization in free recall. *J. Exp. Psychol. Learn. Mem. Cogn.* **50**, 1035–1068 (2023).
11. Healey, M. K., Long, N. M. & Kahana, M. J. Contiguity in episodic memory. *Psychon. bulletin & review* **26**, 699–720 (2019).
12. Lohnas, L. J., Polyn, S. M. & Kahana, M. J. Expanding the scope of memory search: Modeling intralist and interlist effects in free recall. *Psychol. Rev.* **122**, 337–363 (2015).
13. Luhmann, C. C. & Rajaram, S. Memory transmission in small groups and large networks: An agent-based model. *Psychol. Sci.* **26**, 1909–1917 (2015).
14. Raaijmakers, J. G. & Shiffrin, R. M. Search of associative memory. *Psychol. Rev.* **88**, 93–134 (1981).
15. Mannering, W., Rajaram, S. & Jones, M. N. Towards a cognitive model of collaborative memory. In *Proceedings of the Annual Meeting of the Cognitive Science Society*, vol. 43, 959–965 (2021).
16. Murdock, B. B. The serial position effect of free recall. *J. Exp. Psychol.* **64**, 482–488 (1962).
